# Validity of BiliDx as a point-of-care bilirubin measurement device to diagnose and monitor neonatal jaundice at Muhimbili National Hospital, an observational study

**DOI:** 10.1186/s12887-024-04565-w

**Published:** 2024-02-13

**Authors:** Pascal Clemence, Robert Moshiro, Karim Manji

**Affiliations:** 1 Department of Paediatrics and child health, Muhimbili University of Health and Allied Sciences, P.O BOX 65001, Dar es Salaam, Tanzania; 2 Department of Paediatrics and Child Health, Muhimbili National Hospital, P.O BOX 65000, Dar es Salaam, Tanzania

**Keywords:** Neonatal jaundice, Total serum bilirubin (TSB) and BiliDx

## Abstract

**Background:**

Neonatal jaundice is a condition caused by elevated levels of bilirubin in the bloodstream. Laboratory determination of serum bilirubin concentration by total serum bilirubin (TSB) test is still considered as gold standard for clinical guidance and practice. In developed countries, diagnosis of neonatal jaundice is shifting towards point-of-care medical devices. BiliDx is a device developed to allow a fast, blood-based determination of bilirubin levels at the point of care. This study aimed to determine the accuracy of the BiliDx device relative to a standard laboratory total serum bilirubin to diagnose and monitor jaundice among neonates admitted at Muhimbili National Hospital (MNH).

**Material and methodology:**

This was a prospective hospital-based observational study conducted at the Neonatal Ward – MNH, Dar-es-Salaam, Tanzania from November 2022 to January 2023. A total of 180 neonates admitted at the neonatal ward with jaundice and whose parents consented were enrolled in the study. Blood samples were collected; 2 ml of venous blood into the vacutainer bottle for standard laboratory measurement of total serum bilirubin (TSB) and 25µL blood collected into a transfer pipette tube and applied to BiliDx. STATA version 15.1 was used for data analysis.

**Results:**

Out of 180 neonates, 39.4% (71/180) had birth weight between 1500 − 2499.9 g, approximately 2/3rd (120/180) were preterm, 92/180 (51.1%) were males and 100/180 (55.6%) were undergoing phototherapy treatment the moment sample taken. The mean bilirubin concentration was 92 mmol/l for BiliDx and 118 mmol/l for standard laboratory TSB. The minimum and maximum values obtained with BiliDx were, 3.4 and 427.5 mmol/l respectively, compared with 10.7 and 382.1 mmol/l using standard laboratory TSB. A linear relationship and correlation coefficient of 0.8408 (*p* = 0.000) between BiliDx and standard laboratory TSB was found. The regression analysis showed the presence of constant error [coefficient of BiliDx/slope = 0.91, 95% CI (0.82–0.99), *p* = 0.000] and random error exclusively [coefficient of constant/y-intercept = 48.52, 95%CI (37.70-59.34), *p* = 0.000]. The Bland–Altman plot showed an acceptable mean difference of 39.1mmol/l, limits of agreement of -48.3mmol/l to 126.4mmol/l, and 179 points (179/180 = 99.4%) lying inside the limits of agreement.

**Conclusion:**

The results support the use of BiliDx for rapid and accurate testing of elevated levels of bilirubin in the bloodstream among neonates since 99.4% of the differences between BiliDx and standard laboratory TSB lie between the lines of agreement.

**Supplementary Information:**

The online version contains supplementary material available at 10.1186/s12887-024-04565-w.

## Background

Neonatal jaundice, is a condition caused by elevated levels of bilirubin in the bloodstream that presents with yellowish discoloration of sclera and skin. It results from the breakdown of red blood cells. Usually, it shows a cephalocaudal progression starting on the face and then the chest, abdomen, and feet as serum levels increase [1]. It affects approximately half of all newborns in their first week of life and may put them at risk for bilirubin-induced mortality or long-term neurodevelopment impairments, the vast majority of which occurs in low-resource settings [2]. When identified, jaundice is easily treated using blue-light phototherapy [3].

Early diagnosis and accurate testing for elevated serum bilirubin in the first days of life, followed by appropriate treatment with phototherapy, is crucial to prevent brain damage in infants. In developed countries and high-resource settings, neonates with elevated serum bilirubin levels are identified through routine point-of-care bilirubin measuring devices and hospital laboratory testing [4]. However, in low-resource settings, jaundice regularly goes undetected due to a lack of proper and accurate diagnostic tools to measure bilirubin levels [5]. Common methods for evaluation of neonatal jaundice include visual examination (Kramer’s grading system), transcutaneous and serum bilirubin levels. The first two are used for screening or initial assessment and the latter is used for evaluation of severity as well as monitoring progress and response to treatment [6].

Serum bilirubin concentration is the gold standard reference for determining and monitoring the management of neonatal jaundice [7]. It is done by laboratory-based colorimetric assay, which requires large blood volumes and a fully-equipped laboratory often beyond the reach in low-resource settings and for those who can afford do face many challenges both to run the test and obtain results within a meaningful timeframe [5]. While visual assessment by Kramer zone scores poorly correlates with serum bilirubin concentration, transcutaneous bilirubinometers (TcB) have proven to be an alternative to invasive blood sampling [6]. However, TcB may under- or overestimate bilirubin levels and, as they assess extravascular bilirubin, are unreliable if the neonate has received phototherapy treatment [8]. The ideal solution in a low-resource setting would be a reliable point-of-care test that can test serum bilirubin both before and during phototherapy treatment.

The NEST360 (Newborn Essential Solution and Technology) is an ongoing project in 7 hospitals in Tanzania, partnering with the government and other developing partners. The project’s bundle of technologies includes equipment for hydration, nutrition and drug delivery, jaundice management, respiratory support and thermal management. The program supplied point-of-care bilirubin devices to MNH neonatal unit, one of the seven NEST360 sites [9]. However, it is the first time such a device has been used in our setting and there is no local data to show how it compares to the gold standard of serum bilirubin. Therefore, this study aimed to determine whether BiliDx correlates with standard laboratory TSB in measuring serum bilirubin levels among neonates admitted with jaundice.

## Methodology

### Study design

This was a prospective hospital based observational study conducted at neonatal unit, MNH. MNH is a national tertiary level referral hospital receiving patients mainly from the 5 municipalities in Dar es Salaam region (Ilala, Kinondoni, Kigamboni, Ubungo and Temeke). The hospital also receives referral cases from the other 26 regions of the country. It serves as a teaching hospital for Muhimbili University of Health and Allied Sciences (MUHAS). On average there are about 6000 deliveries and the neonatal unit receives approximately 6000–8000 admissions per year.

The neonatal unit at MNH is divided into four sub-units: neonatal intensive care unit (NICU) catering for critically ill babies with a 20-bed state capacity, ward 37 which caters for preterm babies with 44 baby cots, Ward 36 which caters for term babies with 67 cots and the Kangaroo Mother Care (KMC) ward, which accommodates 30 mother-baby pairs.

### Study population

The study population included all newborn babies admitted at the neonatal unit with jaundice and those who developed jaundice during their hospital stay.

### Sample size

Sample size calculations for technical validity of BiliDx assumed power of 80%, an effect size of 0.25, and α = 0.0125, yielding a sample size of at least 185 samples that were needed to detect a statistically significant difference in measurement compared to standard laboratory TSB.

#### Inclusion criteria

All neonates admitted with jaundice and those who developed jaundice in the unit during the study period.

#### Exclusion criteria

All neonates whose caregivers refused to consent to the study.

### Study procedure

Prior to the start of the study, the 2 identified research assistants who are medical officers received training on the BiliDx point-of-care device for measuring serum bilirubin. BiliDx is a device developed to allow a fast, blood-based determination of bilirubin levels at the point of care (Figure A). The device uses lateral flow cassettes, whereby a blood sample is transferred onto the cassette (Figures B and C). The lateral flow strip separates the plasma from the corpuscular components of the blood and allows the determination of TSB by reflectance spectroscopy using the reader (Figure D). Each member was required to pass a standard evaluation conducted by the principal investigator in order to operate the device.

After obtaining the informed consent from the mother of the identified neonate with jaundice blood samples were collected at once; a sample of 2mls of venous blood was drawn into the vacutainer bottle for routine standard laboratory measurement TSB level done by DxC800 using a diazo reagent. Another sample of 25µL of blood was collected into a transfer pipette (capillary tube) and then applied to a test strip inserted in the BiliDx device reader at the bedside.

The decision to take a blood sample for bilirubin measurement was at the clinician’s discretion and was based on the Tanzanian neonatal care guidelines for management of newborn infants with hyperbilirubinemia.

### Data collection, management and analysis

Research assistants recorded patient information and results from both BiliDx and standard laboratory TSB on a standardized patient monitoring form. Socio-demographic, clinical information and laboratory results of participants were crosschecked and coded before being entered into computer software. Data were edited, cleaned, entered and analysed using STATA version 15.1. Categorical data presented in the form of frequency distribution and proportion. The correlation between the gold standard measurement and the tool under test was calculated using Pearson correlation. Then, simple linear regression was performed and a scatter plot was drawn to assess the linearity of the two bilirubin tests and the presence of proportion, constant and random errors. Finally, the Bland Altman plot was drawn to assess the agreement between the two tests.

## Results

### Social demographics and clinical characteristics of the study participants

A total of 180 neonates were recruited, 39.4% (71/180) had birth weight between 1500 and 2499 g, 33.3% (60/180) were born at term and 51.1% (92/180) were males. More than half 100/180 (55.6%) of the neonates had started phototherapy treatment at the time of study (Table 1).

**Table 1:** Social demographics and clinical characteristics of the study participants

Variable	Frequency *n* = 180	Proportion (%)
**Birth weight in grams**		
< 1000 g	4	2.2
1000 -1499.9 g	44	24.4
1500 − 2499.9 g	71	39.4
2500 – 4000 g	53	29.4
> 4000 g	8	4.4
**Gestation age in weeks**		
Extreme preterm (< 28)	4	2.2
Very preterm (28–31)	47	26.1
Moderate preterm (32–33)	36	2.0
Late preterm (34–36)	33	18.3
Term (> 37)	60	33.3
**Sex**		
Male	92	51.1
Female	88	48.9
**Phototherapy treatment**		
Yes	100	55.6
No	80	44.4

### Validation of the accuracy of BiliDx to measure bilirubin concentration in neonates

The mean bilirubin concentration was 92 mmol/l and 118 mmol/l for BiliDx and standard laboratory TSB respectively. The minimum and maximum bilirubin values obtained with standard laboratory TSB and BiliDx were 3.4 and 382.1 mmol/l and 10.7 and 427.5 mmol/l respectively.

**Fig. 1:**
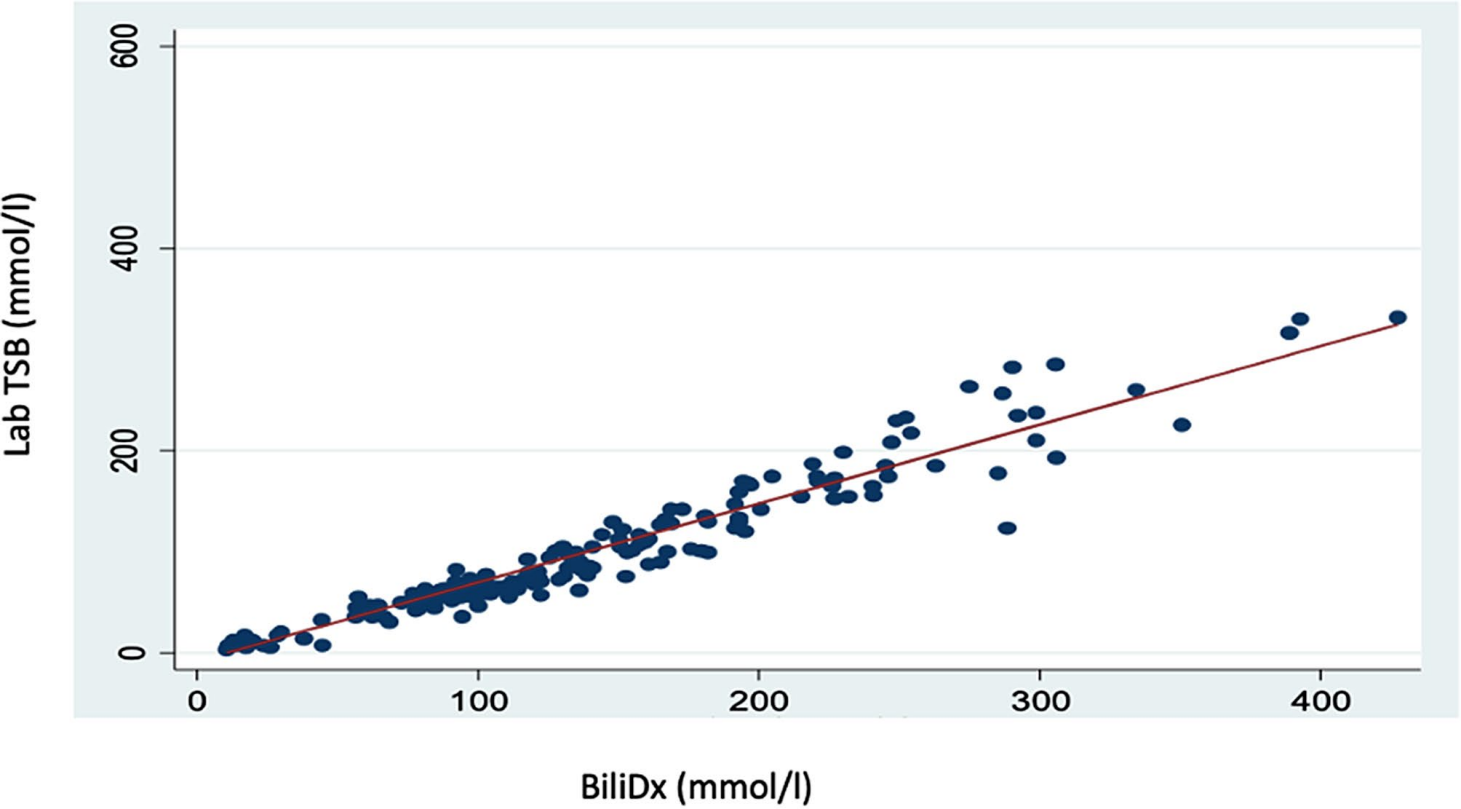
Scatter plot between BiliDx and standard laboratory TSB

The correlation coefficient (r) between BiliDx and standard laboratory TSB was found to be 0.8408 (*p* = 0.000). A linear regression analysis showed a linear relationship between these two bilirubin tests with a positive correlation (Fig. 1). This regression analysis indicated the presence of constant error [coefficient of BiliDx/slope = 0.91, 95% CI (0.82–0.99), *p* = 0.000] during testing the neonates’ bilirubin. Furthermore, regression analysis indicated the absence of proportional error during bilirubin testing [coefficient of constant/y-intercept = 48.52, 95%CI (37.70-59.34), *p* = 0.000] (Table 2).

**Table 2:** Linear regression results

	Coefficient	t	*P* value	95% CI
**BiliDx (slope)**	0.91	20.78	0.000	0.82–0.99
**Constant (y-intercept)**	48.52	8.85	0.000	37.70- 59.34

### Usability of BiliDx to measure bilirubin concentration in neonates

**Fig. 2:**
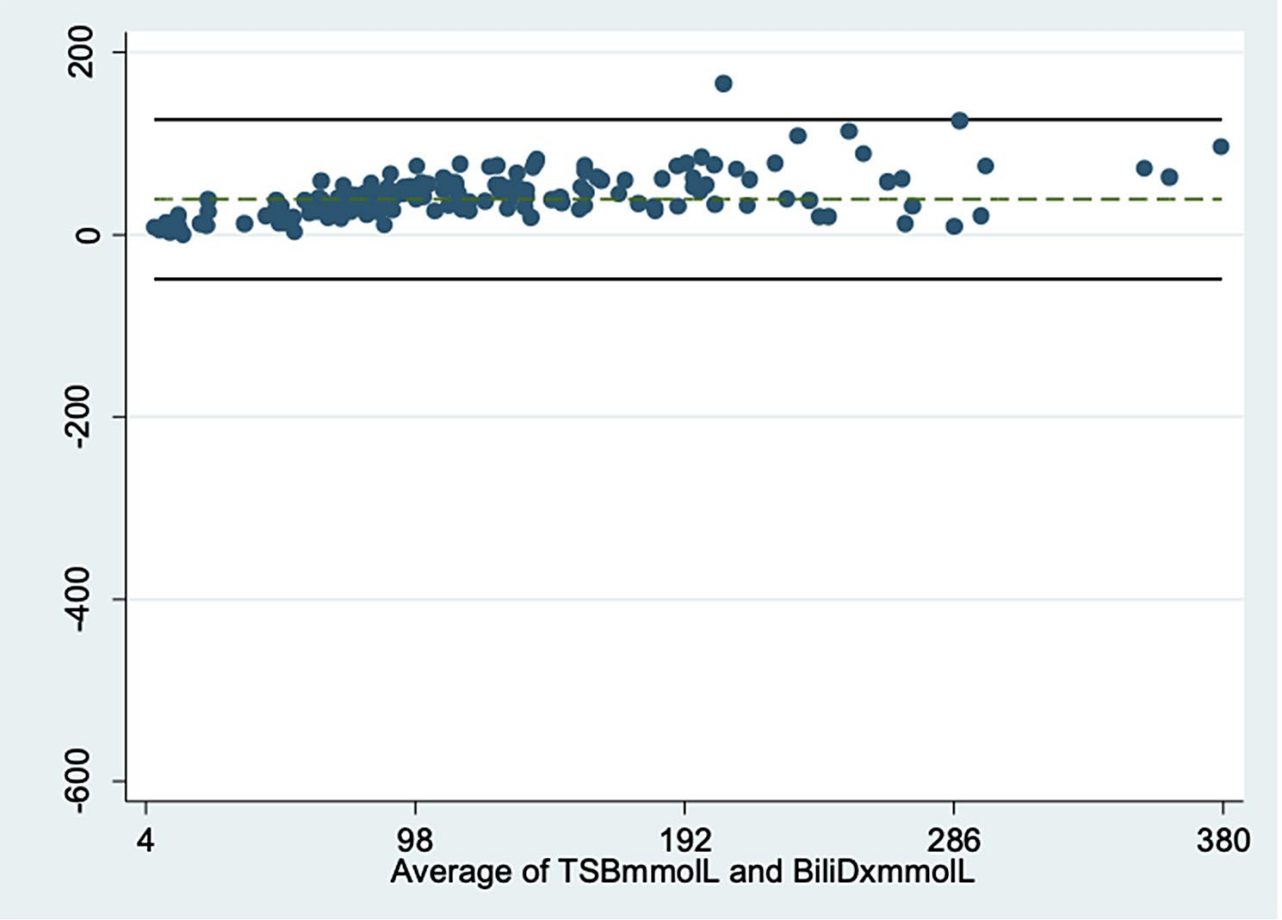
The Bland–Altman plot between differences and average BiliDx measurements (mmol/l) and standard laboratory TSB (mmol/l)

The Bland–Altman plot of the BiliDx measurements (mmol/l) against routine laboratory bilirubinometer TSB showed an acceptable mean difference of 39.1mmol/l with the limits of agreement of -48.3mmol/l to 126.4mmol/l. The maximum observed difference was − 533.1µmol/l with 179 points lying inside the limits of agreement (179/180 = 99.4%) (Fig. 2). Based on the usability decision depending on inherent imprecision as previously described [10], the two bilirubin tests agreed on each other since 99.4% of the differences between BiliDx and TSB lie between the lines of agreement.

### Time to results

The mean duration for obtaining TSB results (Table 3) that included mean time to specimen collection, posting laboratory requests, sample processing, and results verification was 20 h for TSB and 10 min with BiliDx.

**Table 3:** Mean time to results

Type of test	Mean time to results	Range
Laboratory TSB	20 h	16.2–24.4 h
BiliDx	10 min	8–12 min

## Discussion

This study determined the accuracy and usability of a new point-of-care device called BiliDx to measure bilirubin concentration in neonates. The current study reported a correlation coefficient of 0.91 between BiliDx and standard laboratory TSB with an acceptable mean difference of 39mmol/l. of which 99.4% of the differences between BiliDx and standard laboratory TSB lie between the lines of agreements Furthermore, the mean time to results was 10 min compared to 20 h of laboratory TSB.

A similar correlation was reported by Shapiro et al. (2021) who compared BiliDx with a standard laboratory bilirubinometer and transcutaneous bilirubinometer in Malawi [11]. Other studies in Denmark, Italy, USA, Germany and Toronto reported a slightly smaller coefficient [12–17], while a high correlation of 0.961 and 0.86 were seen in Egypt and Italy respectively [18, 19]. These variations in correlation might be explained by a variety of factors including type of device used, time of sample collection, variation in sample collection techniques including whether samples were protected from light or whether sample collection was before or during phototherapy. The majority of our neonates were under phototherapy. Care was taken in our study to ensure phototherapy lights were turned off during collection and the sample was protected from light during the collection and in the laboratory.

In this study, a linear relationship with slope and y-intercept not equal to 1 and 0 respectively was observed between BiliDx and standard laboratory TSB. Our results agree with other point-of-care bilirubin measuring devices reported by Ebbesen et al. (2012) in Denmark, [18], portraying that POC devices for bilirubin measurements are as accurate as standard laboratory TSB.

The current study showed that BiliDx had a good agreement with the gold standard test which is commonly used in the laboratory for the diagnosis of neonatal jaundice. Coda et al. [20] reported the accuracy of their POC device (Bilistick) was also reasonably accurate, negligibly invasive, and could be used for both pre and post-discharge screening of neonatal jaundiced, mostly in low-resource settings. Agreement of the other point-of-care devices with standard laboratory tests has been also reported in Napoli-Italy [19]. High performance of TSB POC tests was also seen in a study done in a low-resource setting by Keahey et al. [5] whereby their device (BiliSpec) performed well compared with laboratory measurement of total bilirubin with all samples being within 3.0 mg/dL (51.3 mmol/l) of the laboratory reference standard. It was seen to offer a simple and accurate measure of TSB to improve diagnosis and monitoring of neonatal jaundice.

Furthermore, we report a short turnaround time for BiliDx compared to standard laboratory TSB (Table 3). As expected, point-of-care devices do provide results quicker. However, the clinical implication of this finding together with the previous reports [11, 20], provides an opportunity for early initiation of treatment, and subsequently effecting the course of jaundice. The process of sample collection was less cumbersome with BiliDx as compared with standard laboratory tests. The use of heel prick for sample collection can be done easily and more efficiently than venipuncture, a process needed to get enough sample for standard laboratory testing. We believe that BiliDx is a simple, accurate and affordable alternative to the current available techniques to measure TSB in low-resource settings. Its short turnaround time does not compromise quality, hence improving patient outcomes in situations where a quick bilirubin estimation is required for decision-making.

## Conclusion

BiliDx showed to have short mean time to results and a high correlation coefficient indicating its beneficial use as a point of care bilirubin measuring device in areas where there is no laboratory TSB and/or where there is high turnaround time for test results. Therefore, this supports the use of BiliDx for rapid and accurate testing of elevated levels of bilirubin in the bloodstream and for monitoring treatment progress while on phototherapy among neonates with jaundice.

### Electronic supplementary material

Below is the link to the electronic supplementary material.

Supplementary Material 1

Supplementary Material 2

Supplementary Material 3

Supplementary Material 4

## Data Availability

The data from this study can be obtained upon request from the first author, Dr. Pascal Clemence. email: pascalclemence@gmail.com.

## Abbreviations

EPT: Extremely preterm infant
GCP: Good clinical practice
LBW: Low Birth Weight
MNH: Muhimbili National Hospital
MUHAS: Muhimbili University of Health Allied Sciences
NCU: Neonatal Care Unit
NICU: Neonatal Intensive Care Unit
SDGs: Sustainable Development Goals
TcB: Transcutaneous bilirubinometer
TSB: Total serum bilirubin
VPT: Very preterm infant
WHO: World Health Organization
