# Supplementary figures and images for: Validity of BiliDx as a point-of-care bilirubin measurement device to diagnose and monitor neonatal jaundice at Muhimbili National Hospital, an observational study
Source: BMC Pediatr. 2024 Feb 13;24:114. doi: 10.1186/s12887-024-04565-w (PMC10863160; doi:10.1186/s12887-024-04565-w)

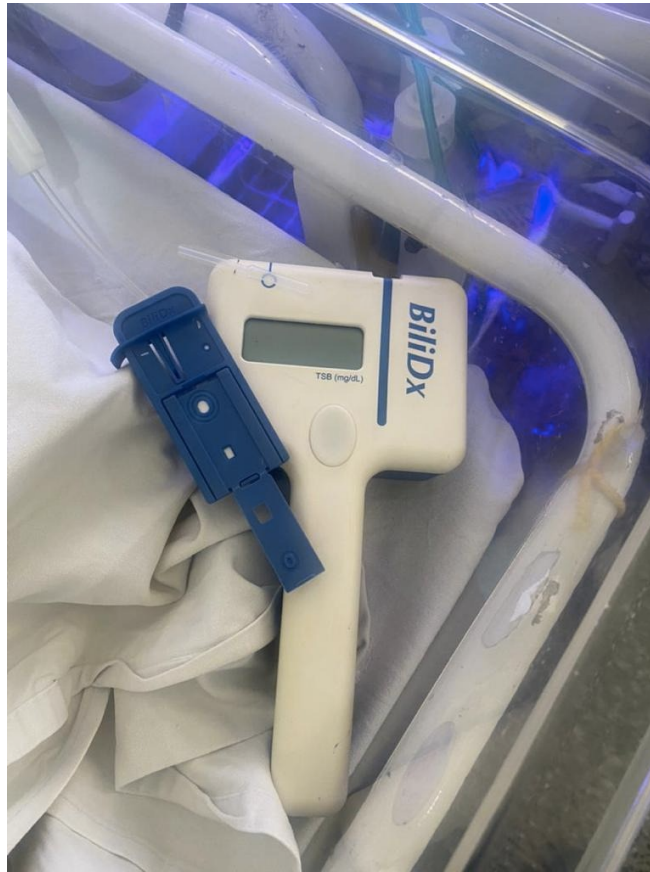

Figure A: BiliDx used during the study

Supplement: Supplementary file 1 — Supplementary Material 1 [file 12887_2024_4565_MOESM1_ESM.pdf]

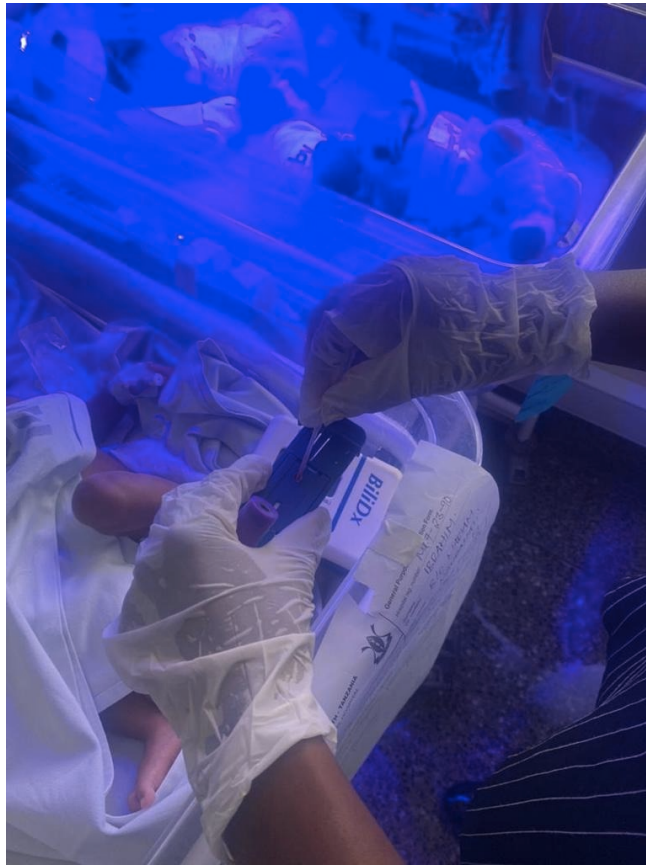

Figure B: Use of the pipette to add sample to the cartridge

Supplement: Supplementary file 2 — Supplementary Material 2 [file 12887_2024_4565_MOESM2_ESM.pdf]

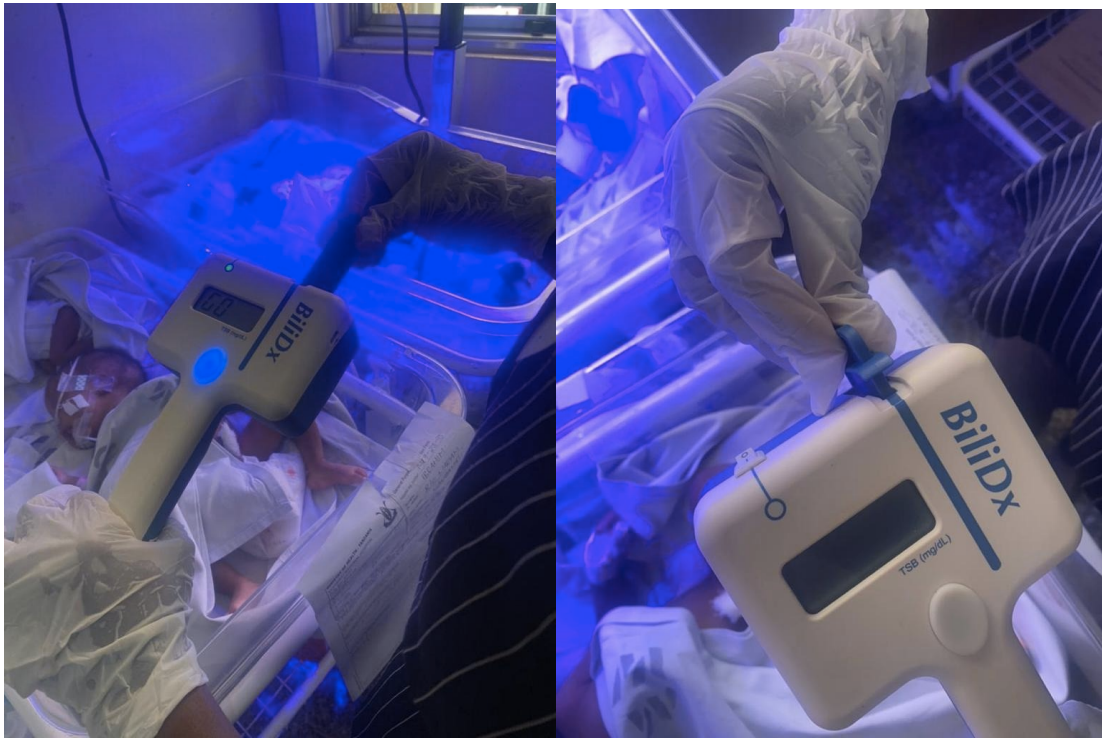

Figure C: Insertion of the cartridge into the reader

Supplement: Supplementary file 3 — Supplementary Material 3 [file 12887_2024_4565_MOESM3_ESM.pdf]

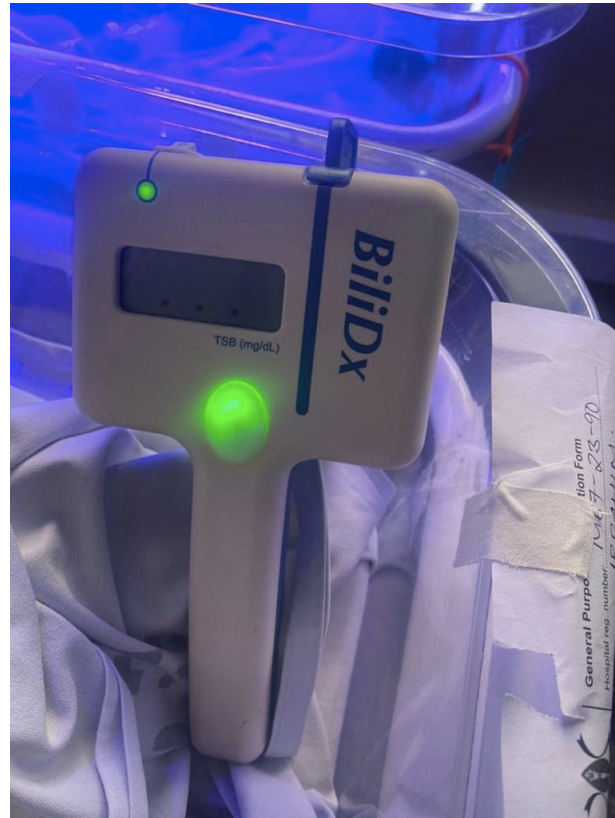

Figure D: Ongoing point-of-care bilirubin measurement

Supplement: Supplementary file 4 — Supplementary Material 4 [file 12887_2024_4565_MOESM4_ESM.pdf]
